# Of the Drug Curative

**Published:** 1890-02

**Authors:** P. P. Wells


### OF THE DRUG CURATIVE.

Dr. P. P. Wells.

Communicated by C. Carleton Smith, M. D., Philadelphia, Pa.

(Continued from p. 10.)

We have, at present, no other concern with this process than to present it as additional evidence of the truth as to the nature of the curative principle which we advocate. In order to this, it was incumbent, first, to show that the process itself is a fact. The experience of Channing is sufficient for this. But, we may add, the fact has been known, and is now fully accepted by many of the best minds in our school of the profession.

In prosecuting the argument from the facts here presented, it is pertinent to inquire what passed from the globules contained in the phial, supposed to be medicated, to those added, which were not, by which these last received the power, like those previously contained, to affect the organism of the sick. Was it matter? Is there, in the world of matter, anything like this? On the other hand, is there not an equivalent in the transmission of *force* from steel magnets to similar bars, not magnetic, by *contact* alone, till all alike contain the magnetic force, though the original magnet, like the original globules, loses nothing of its power by imparting to the new-made magnets a force similar to its own. Medication by contact, then, becomes little, if at all, less than a demonstration of the dynamic nature of the principle under consideration. That the power which so passed was matter, though we always use the word with reluctance, we have no hesitation in saying is *impossible.*

Argument for the dynamic nature of the curative principle may be strengthened by the fact of the suddenness of its action. In this respect the considerations presented from the instantaneous action of the miasmata, may be applied with so much the more effect to the power we are now considering, as it is, in some respects, brought more readily and completely under our observation.

The precise time of the reception of the medicinal agent may always be exactly known, while in the case of the miasm this is not always easily brought to a fixed point. Then, the effects of drug agents have been more thoroughly studied, and are better known than those of the miasmata, especially in those minute details which characterize the action of drugs. So that in all that wherein we are able to give increased certainty to the points, as we present them in relation to the subject under consideration, by so much is the force of the argument greater here than when applied to the miasms. Two cases will be presented as illustrations of this immediate action of the drug power, both of which were under the immediate observation of the writer, and both are made up of elements which, it is believed, place them beyond reach of even the most captious caviler. They are selected from a multitude of similar cases for these two reasons: The first is memorable in the experience of the writer, it being the result of his *first* attempt at a homœopathic prescription for a patient. It was the first experiment in a series, which the importunity of a valued friend had extorted the promise, a very reluctant one, that he would make.

The whole intention on the part of the writer was, in these experiments, to prove for himself and his friends *that there was nothing in Homœopathy.*

If it be objected that this was altogether unfair, and unbecoming the importance of the subject, it is admitted to be true. He *intended* to prove the worthlessness of the whole system, and had no doubt of his success. Here is his first attempt and its result. It will be seen that Homœopathy had nothing to expect from him, except what could be extorted by the most apparent and stubborn fact.

The experiment was made with the despised "*globules,*" and no one certainly ever held them in greater contempt.

It was on a patient verily believed to be incurable of the particular trouble for which these globules were given. The writer had tried his best, according to the maxims and practice of the school in which he had been educated, for months to relieve the poor sufferer, without the slightest success. It was just because he thought the case incurable that it was taken for experiment. Here, of course, would be a failure.

He wished for no success. Then the patient was poor, ignorant, and black. She had never heard the word "Homœopathy," and so the wonderful effects of imagination would be escaped, which, with many others, he thought to be the efficient agent in all the so-called homœopathic cures. She had formerly been the slave of an old-school doctor.

This person, now about forty-eight years of age, when a child fell from a tree, and struck her side on the top of a board fence, breaking several of her ribs. She had from that time, occasionally, attacks of pain at the points of fracture, increasing in frequency and severity as she grew older, till, at the time she came under the care of the writer, it had become permanent, and yielded to none of the many expedients resorted to, as before stated, for a number of months. Indeed, she grew worse. The seat of pain was so sensitive, she positively refused to allow it to be touched. Here was the case of more than forty years' standing. In this state she was handed a powder of fine sugar, in which were *concealed* six globules of Arnica of the sixth potency. She was directed to take the powder dry, on the tongue. This she did at eleven o'clock A. M., in the absence of her medical attendant. When seen the next day at nine o'clock A. M., she appeared in great terror. She seemed as much frightened as any person I ever saw. She would not let me come near her, but kept herself in the extreme opposite side of the room, and repeated more than once, addressing her physician, "You meant to kill me, you gave me Mercury. I know Mercury. I lived with Dr. Hazard," etc. After quieting her apprehensions—not an easy matter—she was asked if she had taken the powder. She said she had; and the manner of her reply was just that of a person who regards himself as having just been made the subject of a joke of a severe and unwarranted character.

She was soon pacified, and then was asked what was its effect. "Effect! *It was from there to there,*" passing her finger from her tongue to the spot in her left side, the seat of the old injury, and recent pain "*like lightning.*" "*You gave me Mercury. I know you did.*" And this thought renewed her terror. She could not overcome the first impression that she had been poisoned. In a little time, however, she answered to the question, "What happened then?" "*Why, the pain and soreness went all right out of it.*" This was true, and they never returned. The side might now be handled with the utmost freedom without giving pain. The person, next to herself, most astonished was her physician. Rosana was, after this, a servant in his family till near the time of her death, which resulted from disease of the heart, and he knows the old trouble did not return. Of the suddenness and completeness of the action of this dose, there was no possible chance for doubt. That it was, literally, as quick as lightning, she stoutly maintained, that she was really cured of her great pain, could not be denied. And now, the only question we have with the case, is—*what was it* that passed from the tongue to the side so suddenly as to give this great alarm to this poor, ignorant creature, and so rapidly to cure this great pain? Was this matter? The suddenness of its action proves that it was not.

